# Follicular dendritic cell sarcoma with atypical features surrounding undescended testis: description of a rare case

**DOI:** 10.1186/s12957-015-0494-9

**Published:** 2015-02-21

**Authors:** Alberto Testori, Stefano Meroni, Piergiuseppe Colombo, Stefano Fiori, Emanuele Voulaz, Marco Alloisio

**Affiliations:** Department of Thoracic and General Surgery, Humanitas Research Hospital, Via Manzoni, 56, 20089 Rozzano Milan, Italy; Division of Breast Radiology, European Institute of Oncology, Via Ripamonti, 435, 20141 Milan, Italy; Department of Pathology, Humanitas Research Hospital, Via Manzoni, 56, 20089 Rozzano Milan, Italy

## Abstract

Dendritic cell tumors are extremely rare neoplasms and occur both in nodal and extranodal sites. We report a case of an intra-abdominal follicular dendritic cell sarcoma (FDCS). The aim of this study is to describe histological, immunohistochemical, and ultrastructural features of FDCS in order to better define an abdominal mass with unusual immunophenotype and atypical clinical and radiological presentation.

## Background

Dendritic cell tumors are rare entities which have been previously described by a variety of terms such as lymphoma, sarcoma, or histiocytic neoplasms, reflecting the controversy that surrounds these tumors [1]. Dendritic cells originate from a heterogeneous group of non-lymphoid, nonphagocytic immune accessory cells in lymphoid districts or in non-lymphoid organs. Currently, these elements are classified as follicular, interdigitating, Langerhans’, and histiocytic/fibroblastic cells [2].

Dendritic cell neoplasms are illustrated by the occasional occurrence of unremarkable clinical and radiological features making a differential diagnosis challenging.

However, radiological and histological features can mimic a wide variety of other tumors and tumor-like lesions, among which mesenchymal tumors, spindle cell carcinomas, and inflammatory pseudotumors are the main differential diagnoses. Even though imaging plays a fundamental role in the first diagnostic approach, histological examination, immunohistochemical analysis, and possibly electron microscopy provide the final diagnosis.

Here, we report clinicopathological, immunophenotypic, ultrastructural, and imaging features of a case of intra-abdominal follicular dendritic cell tumor.

## Case presentation

A 46-year-old male was referred to our hospital with acute onset right flank abdominal pain with no other associated symptoms. Physical examination revealed right upper quadrant/flank tenderness, oral pemphigus, and undescended testis. Clinical and oncological history was unremarkable. The patient underwent a computed tomography (CT) examination to further evaluate the abdominal pain during emergency room work-out.

**Figure 1:**
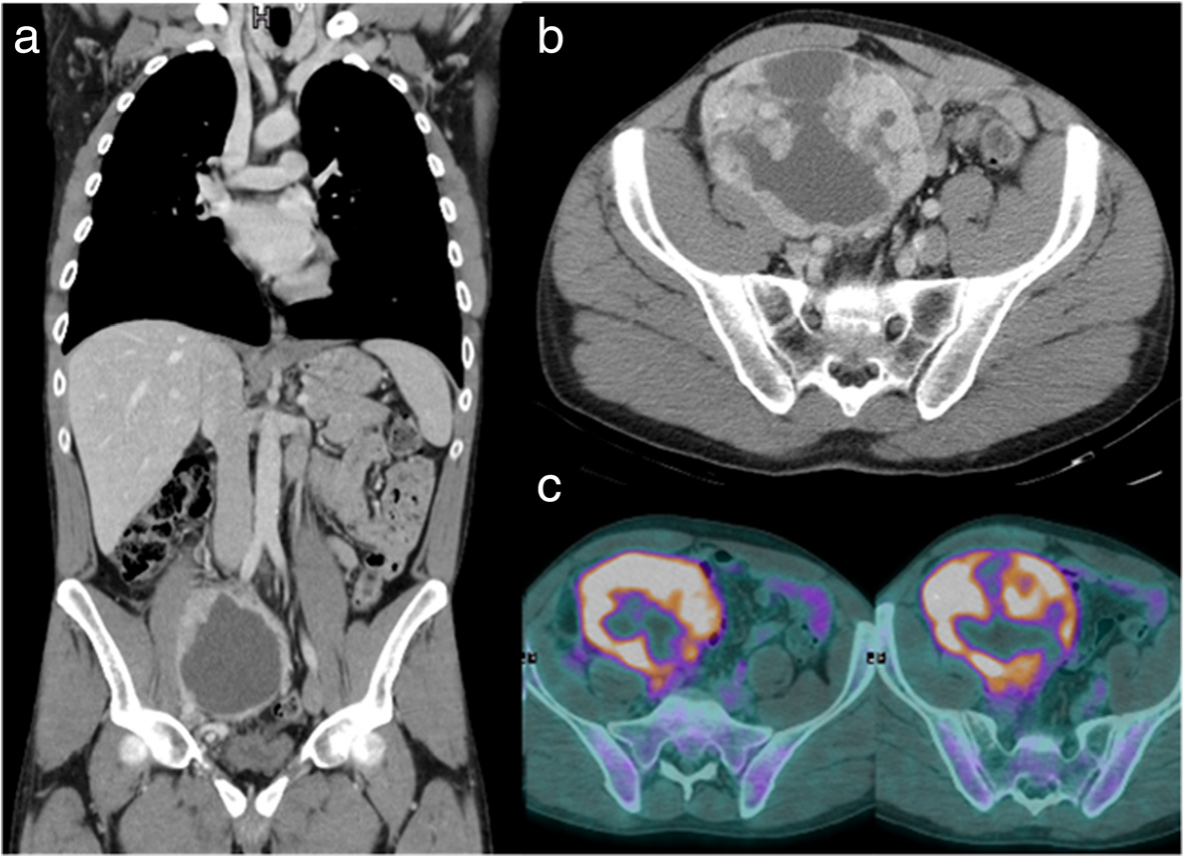
**Coronal CT imaging.** Coronal CT imaging showed a large mass occupying most of the lower portion of the right abdomen with a dyshomogeneous density due to cystic and necrotic areas and no infiltration of the surrounding tissues **(a)**. The tumor is located near the omolateral outer iliac vein as well with secondary dislocation and suspect infiltration **(b)**. In the PET/CT imaging, the central photopenic portion of the mass might reflect necrotic process **(c)**.

The CT images showed a 12-cm rounded mass of heterogeneous solid tissue localized in the lower abdomen between intestinal loops; no enlarged lymph nodes were observed. Coronal CT imaging showed a large mass occupying most of the lower portion of the right abdomen with a dyshomogeneous density due to cystic and necrotic areas and no infiltration of the surrounding tissues (Figure 1a). There were no calcifications in the mass; liquid in the pelvic cavity was detected. The tumor involved the omolateral outer iliac vein as well as secondary dislocation and suspect infiltration (Figure 1b).

A preoperative positron emission tomography (PET/CT) scan was performed and showed a large ^18^ F-FDG (fludeoxyglucose ^18^ F) avid mass in the lower right abdomen corresponding to the CT finding. The central photopenic portion of the mass might be reflecting necrotic processes (Figure 1c).

In order to characterize the lesion, we performed biopsies of the tumor using a CT-guided 18-gauge biopsy, which revealed a poorly differentiated malignant neoplasm, not otherwise specified. The small amount of tissue was not adequate for further analysis.

Patient was in good general health conditions, laboratory tests were unremarkable, and the presurgical work-out (electrocardiogram and chest X-ray) showed no contraindications to open major abdominal surgery.

**Figure 2:**
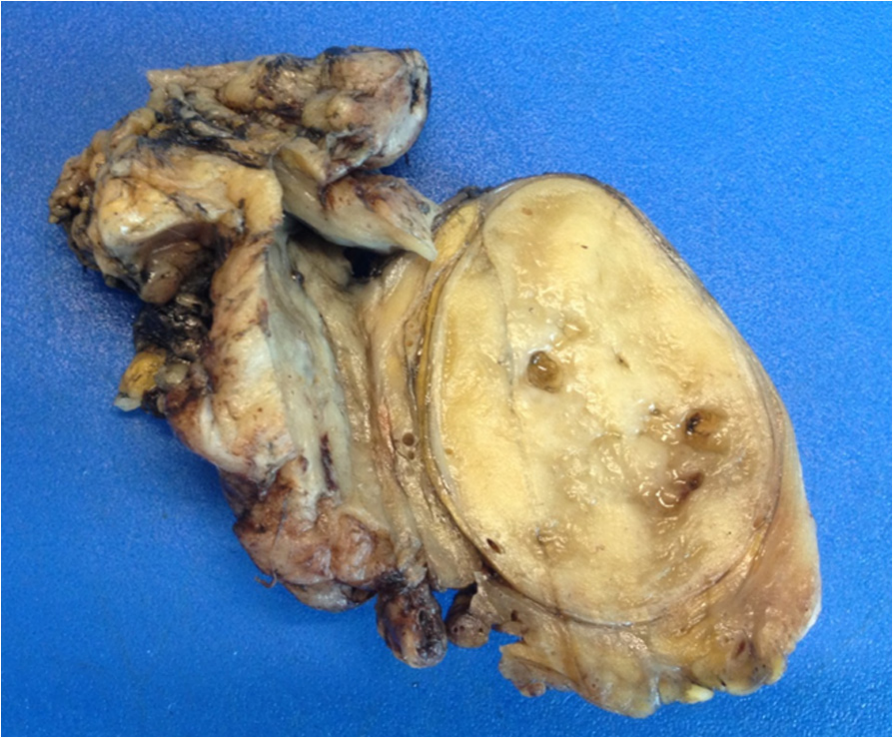
**Gross specimen showed encapsulated solid mass, yellowish-grey to tan, with focal microcystic spaces, with scattered hemorrhage.** An atrophic testis, free of tumor, was also evident.

**Figure 3:**
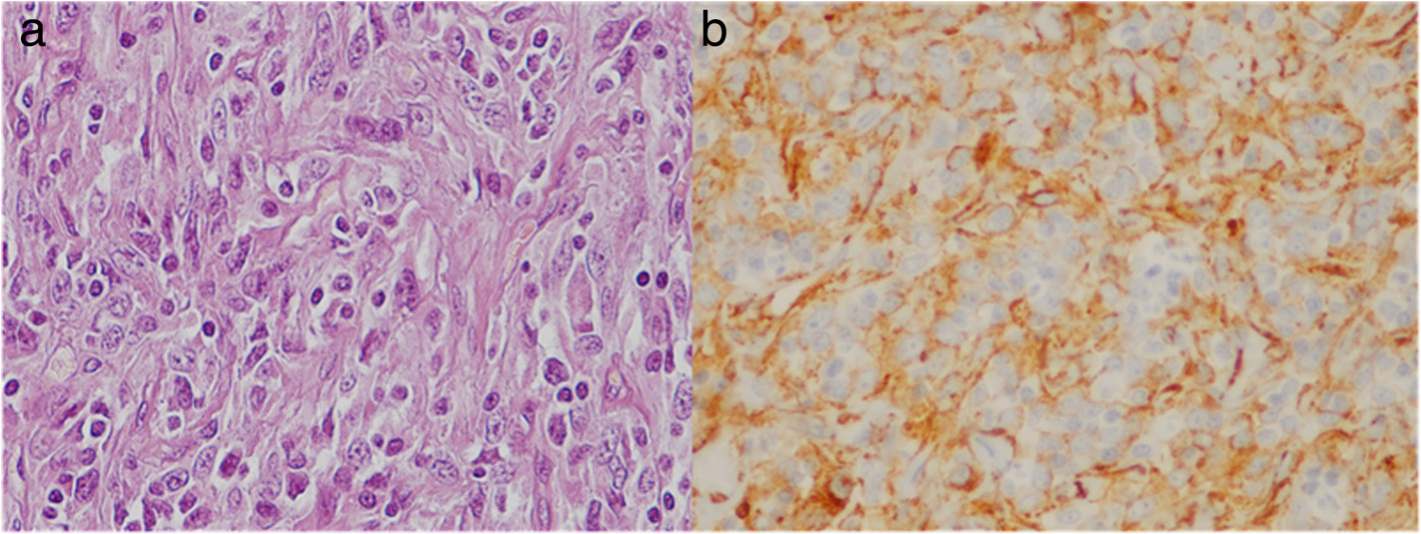
**Histologic appearance and immunohistochemical findings of the FDC. (a)** Histologic appearance of the follicular dendritic cell sarcoma showing characteristic spindle and ovoid tumor cells with elongated nuclei and prominent nucleoli; note the presence of numerous lymphocytes (hematoxylin and eosin staining, original magnification ×400). **(b)** Immunohistochemical findings of the FDC sarcoma; note the expression of tumor cells for H-caldesmon in scattered cells (IHC staining, original magnification ×400).

The patient underwent resection of the mass through a midline laparotomy incision. A tangential surgical resection of the venous vessel was necessary to ensure a correct oncological debulking, since the right outer iliac vein infiltration could not be excluded by preoperative CT imaging. The resection margins of the lesion and the dissected lymph nodes were free of cancer cells, demonstrating false-positive findings on imaging. Gross examination of the specimen showed a 10-cm, encapsulated, nodular mass, adjacent to a 1.8-cm cryptorchid testis with no tumoral infiltration. The mass had a firm, homogeneous grey-tan cut surface, with few (<5%) areas of necrosis and hemorrhage (Figure 2). After an extensive sampling of the specimen, light microscopy revealed ovoid to spindle cells arranged in a storiform proliferation, with a clustering pattern of growth. This feature was embedded in a heavy lymphoid stroma and a diffuse, thin-band interstitial fibrosis. The neoplastic cells were characterized by an abundant, eosinophilic cytoplasm, a finely dispersed chromatin, and one or few evident *nucleoli* (Figure 3a). Overall, the cytological features were uniform, with low-grade atypia. Histological examination found scattered bizarre, bi- or multinucleated giant cells. Mitotic count was <10 mitoses/10 HPF and necrosis was focal. The tumor was well-demarcated, without infiltration of the fibrous capsule thickness. These features, along with the massive lymphoid stroma, suggested a nodal structure, although lymphoid follicles were not evident. Taking into consideration these features, we suspected a nodal accessory-cell sarcoma. The neoplastic cells stained strongly and diffusely for CD68 KP-1, and weakly focally for CD45 and H-caldesmon (Figure 3b). S-100 protein was positive in scattered cells, while CD21, CD23, CD35, CD20, CD3, CD30, ALK-1, cytokeratin AE1/AE3, epithelial membrane antigen (EMA), smooth muscle actin, desmin, calponin, CD31, CD34, HMB-45, and melan-A were negative. Additionally, the follicular dendritic cell markers CD21, CD23, and CD35 confirmed the absence of residual germinal centers. Ki67/MIB-1 proliferation index was low (<5%). These morphological and immunophenotypical aspects were highly suspicious for a histiocytic/dendritic tumor; at the same time, no feature was specific for a subtype. For this reason, an ultrastructural study was considered necessary, and no further immunohistochemical evaluation was performed.

**Figure 4:**
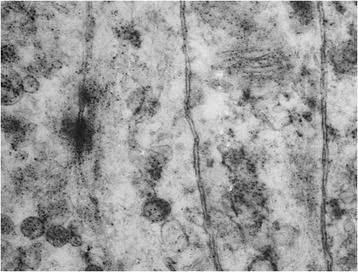
**Electron microscopy.** Electron microscopy analysis showed interdigitating cell processes and desmosome junctions suggestive of a follicular dendritic histogenesis of the tumor.

Electron microscopy analysis revealed that tumor cells were suitable for a follicular dendritic cell histogenesis (interdigitating cell processes and desmosome junctions) (Figure 4).

According to the 2008 WHO Classification of Tumours of Haematopoietic and Lymphoid Tissues, the diagnosis was nodal follicular dendritic cell sarcoma with atypical immunophenotype and myogenic/myofibroblastic differentiation (H-caldesmon +) [3].

No residual disease was found in the testis and surgical margins. Testicular parenchyma showed atrophy and hyalinization of the tubules, with halt of maturation sequence in germ lines.

Postoperative course was uneventful, and the patient was discharged on the third postoperative day. After multidisciplinary team discussion, no adjuvant treatment was performed after the surgery. No recurrence occurred after 1-year follow-up.

## Discussion

Dendritic cell neoplasms are rare tumors that have been previously described by a variety of terms such as lymphoma, sarcoma, or histiocytic neoplasm, reflecting the controversy that surrounds these tumors [1].

Dendritic cells originate from a heterogeneous group of non-lymphoid, nonphagocytic immune accessory cells found in lymphoid and non-lymphoid organs; there are several types of dendritic cells: follicular, interdigitating, Langerhans’, and histiocytic/fibroblastic cells [2].

As previously described by some authors, dendritic cells play a role in antigen capture and presentation, providing structural and functional basis to the biochemical microenvironment within the lymphoid tissue. Due to the variety of dendritic cells and depending on the immunophenotype, various histological subtypes of dendritic cell neoplasms are present [2,4].

Follicular dendritic cell sarcoma (FDCS) is specifically immunopositive to CD21, CD35, and/or CD23, vimentin, fascin, HLA-DR, EMA, D2-40, clusterin, and CXCL13. Immunohistochemical staining in the tumor cells is variously positive for S100, CD68, CD45, and CD20 [5].

The tumor was negative for classical markers CD21, CD23, and EMA, while there was immunoreactivity for H-caldesmon, which could indicate a myogenic/myofibroblastic cell differentiation. This entity could be a variant described in the WHO classification as ‘fibroblastic reticular cell tumor,’ histologically similar to follicular or interdigitating dendritic cell sarcoma, but lacks the immunophenotypic profile of these tumor types (CD21, CD23, or CD35 positivity).

Accordingly, the tumor shows variable immunoreactivity for myogenic markers and CD68 [3]. Prognosis of this variant of dendritic cell sarcoma is similar to the classic type.

The majority of dendritic cell neoplasms are benign/low-grade soft tissue sarcomas, although rare highly malignant cases have been reported [5,6].

The differential diagnosis of FDCS includes other tumors of histiocytic or dendritic origin. The differential diagnosis from other types of tumor is important, since dendritic cell neoplasm has a favorable prognosis whereas other histiocytic/lymphoid tumors, such as histiocytic sarcoma, can be more aggressive. It may be difficult to manage these cases, and immunohistochemical markers (clusterin, CXCL13, FDC markers) may help in making a correct differential diagnosis [7,8].

FDCS has also to be distinguished from neurogenic tumors, germ cell tumors, and mesenchymal tumors such as solitary fibrous tumor, lipoma, epithelioid hemangioendothelioma, and inflammatory pseudotumor.

Usually, dendritic cell neoplasms arise within the lymph nodes, most commonly involving cervical and mediastinal lymph nodes. Dendritic cell neoplasms can arise at extranodal sites; an abdominal presentation, as in our case, is extremely rare and literature reported only few studies. The challenging aspects of our case were the lower abdominal location (into a lymph node near the undescended testis), the clinical and radiological features, and the fact that specimens obtained by CT biopsy were unremarkable.

The majority of patients affected by this disease are asymptomatic. The clinical presentation varies according to the location of the primary tumor. Symptomatic dendritic cell neoplasm typically presents as a painless, slow-growing well-circumscribed mass, and no constitutional symptoms, such as fever, night sweats, and weight loss but in this case the oral pemphigus may be considered as paraneoplastic syndrome. It is well known that oral pemphigus, as in our case, and other cutaneous paraneoplastic syndromes may precede, be concurrent with, or follow the diagnosis of an underlying malignancy.

Usually FDCS and dendritic cell tumors are incidentally discovered as a single intra-abdominal mass identified on a CT scan (incidental finding).

All radiological features reported in literature, particularly those relating to the CT imaging, showed typical morphological aspects of an expansive mass with an increasingly inhomogeneous enhancement, directly proportional to lesion size (due to central necrosis, hemorrhage, and cystic changes with a patchy pattern). Our case complied with these features. There were no obvious pathognomonic radiological signs of dendritic cell neoplasm. Other imaging modalities to investigate the abdominal mass are the magnetic resonance and PET/CT imaging.

The principle of PET scan is based on human tissue uptake of a glucose analog that has been tagged with a positron-emitting isotope (^18^ F-FDG): this process is intensified in malignant cells. In our case, the radiological enlarged abdominal mass was secondary to areas of necrosis (the central photopenic portion on PET scan confirmed by the macroscopic surgical specimen), given the large size of the tumor. Moreover, the area of necrosis was also confirmed by the macroscopic surgical specimen during surgery.

Imaging techniques are fundamental in obtaining a preliminary diagnosis based on morphological and functional features of dendritic cell neoplasm, while the final diagnosis is determined by interventional approaches (biopsy or surgery).

Morphological features, immunohistochemical analysis, and electron microscopy are useful to obtain the final diagnosis [9].

Most of the data on abdominal dendritic cell neoplasm is based on case reports or small case series, and the natural history and response to different therapeutic modalities in this disease have not been well established. To our knowledge, there is no standard treatment algorithm in such cases. Surgical resection remains the cornerstone of treatment, while radiation therapy and chemotherapy in the neoadjuvant, adjuvant, and metastatic setting are yet undefined.

## Conclusions

Intra-abdominal dendritic cell neoplasm is extremely rare.

The pathologist should take this neoplasm into consideration, due to differential diagnosis of unusual spindle cell lesions (such as gastrointestinal stromal tumors or solitary fibrous tumors) with a significant background population of small lymphocytes. Only skilled pathologist should perform differential diagnosis.

Due to its nonspecific histologic appearance, additional electron microscopic studies are generally required to define the correct diagnosis and the histological subtype.

Although surgery, whenever feasible, is the primary treatment, multimodality approach and patient-related treatment should be considered.

Further studies are necessary to manage these particular patients.

## Consent

Patient consent was obtained for this study.
